# Detection and prevention: evaluation of a nurse-led satellite sexually transmitted infection (STI) testing clinic initiative in an Australian correctional centre

**DOI:** 10.1108/IJOPH-09-2024-0053

**Published:** 2025-06-23

**Authors:** Amanda Nichole Balmer, Annette Brömdal, Sonya Osborne, Kathryn Kynoch, Amy Mullens, Leah East

**Affiliations:** Graduate Research School,School of Nursing and Midwifery, University of Southern Queensland – Toowoomba Campus, Toowoomba, Australia; Centre for Health Research, University of Southern Queensland, Queensland, Australia; School of Nursing and Midwifery, University of Southern Queensland – Toowoomba Campus, Toowoomba, Australia; Centre for Health Research, University of Southern Queensland, Queensland, Australia; School of Nursing and Midwifery, University of Southern Queensland – Toowoomba Campus, Toowoomba, Australia

**Keywords:** Australia, Health in prison, Prisoners, Correctional health care, Prison, Sexual health, Infectious disease

## Abstract

**Purpose:**

The purpose of this study was to determine if a satellite sexually transmitted infection (STI) testing clinic increased STI/blood-borne virus testing and detection in a correctional setting in Australia.

**Design/methodology/approach:**

A cohort study of males incarcerated in a correctional centre in Queensland, who voluntarily attended a satellite STI testing clinic over six months. Data were collected on purpose designed data collection sheets. A retrospective medical chart audit was conducted from three-months before commencement of the clinic until the end of the clinic period. Attendance rates for three-months following the clinic’s completion were also considered. Attendance rates, treatment rates, time to treatment, follow-up rates, reinfection rates and client satisfaction were analysed using descriptive statistics, including program sustainability.

**Findings:**

Success of the STI clinic was evidenced by an increase in attendance rates from 32/242 (13.2%) to 242/242 (100%), pre-intervention to the intervention, respectively. Treatment rates increased from 10/242 (4.1%) to 41/242 (16.9%) pre-intervention to intervention, respectively, and an increase in time to treatment from 43.11 (Std. Dev 36.77) mean days pre-intervention to 54.62 (Std. Dev 42.06) mean days during the intervention. Follow-up rates also increased from 5/242 (2.1%) pre-intervention to 24/242 (9.9%) during the intervention. Of the 242 participants, 52 received a positive STI/diagnosis with 44 being diagnosed with hepatitis C. Satisfaction was high with a mean score of 9.7 out of 10 (Std. Dev 0.685). Attendance rates showed no significant difference three-months pre- (*n* = 32) to post-intervention (*n* = 35), however, support for the intervention has continued. Future practice should incorporate satellite STI testing clinics as weekly practice.

**Originality/value:**

To the best of the authors’ knowledge, this is the first study to consider satellite STI testing within the corrections environment in Australia. The study uniquely showcased how the satellite STI clinic achieved increased STI testing attendance rates, treatment rates, follow-up rates and high satisfaction rates.

## Introduction

Multiple barriers affect the health and wellbeing of incarcerated people (Queensland Government, 2020; Australian Institute of Health and Welfare, 2023). More specifically, barriers affecting sexual health include limited access to sexually transmitted infection (STI) prevention, testing and treatment in correctional facilities (World Health Organization, 2024; Balmer *et al.*, 2025; Balmer *et al.*, 2023), which is exacerbated by issues associated with accessing medical departments for consultation resultant of environmental time constraints within custodial settings (Australian Institute of Health and Welfare, 2023). People residing in correctional facilities are disproportionately impacted by STIs and blood-borne viruses (BBVs) including hepatitis C (HCV), hepatitis B (HBV) and human immunodeficiency virus (HIV) (World Health Organization, 2023). Globally HCV affects one in every four incarcerated people (World Health Organization, 2024). Within Australia, 14% of individuals discharging from prison in 2022 self-reported that they were diagnosed with a communicable disease inclusive of STIs/BBVs whilst incarcerated (Australian Institute of Health and Welfare, 2023). Whilst the level of sexual health knowledge held by those that are incarcerated is comparative to those residing in the general community (Malacova *et al.*, 2011), incarcerated populations are disproportionately impacted by STIs/BBVs compared to the general population. This can be attributed to risk-behaviours inherent among incarcerated populations, such as intravenous drug use (henceforth IVDU), tattooing/body piercing and unprotected sex (Brömdal *et al.*, 2019; World Health Organization, 2014). In 2022, during discharge, 14% of males in Australian prisons self-reported that they had injected substances whilst incarcerated, 7.2% took part in body piercing or tattooing (Australian Institute of Health and Welfare, 2023). In the same year, 38% of Australian males entering prison self-reported that they always participated in unprotected sex, and 42% disclosed that they often or sometimes had casual unprotected sex (Australian Institute of Health and Welfare, 2023); supporting the need for preventative measures, such as prophylaxis-based interventions. For example, condom and dental dam distributions have been explored in some states of Australia (Yap *et al.*, 2007, 2010), however, in Queensland (the state where this study occurred), interventions such as these have not been adopted (Hepatitis Queensland, 2024).

According to the Australasian Society for HIV, Viral Hepatitis and Sexual Health Medicine (ASHM) Australian STI Management Guideline for people in custodial settings (2021a), all incarcerated people should be afforded STI and BBV testing during admission. Guidelines also suggest that in addition to testing on entry to prison, regular STI and BBV testing should be offered during the incarceration period (Australasian Society for HIV Viral Hepatitis and Sexual Health Medicine, 2021a), however there is currently no standardised intervention in place to support this. Within community settings, mobile STI testing initiatives, where the STI testing is taken to the individuals, has shown to be successful in reaching at-risk populations (Hesse *et al.*, 2015), yet there is no standard practice within custodial settings across Australia.

### Objective

There is paucity in the literature focused on satellite STI testing clinics, delivered regularly to incarcerated adults. Therefore, a trial nurse-led satellite STI testing clinic was developed and implemented in an Australian correctional centre. This study aimed to determine if the satellite clinic increased STI testing, and detection through clinic attendance rates, treatment rates, time to treatment, follow-up rates and reinfection rates. The study also explored client satisfaction and level of sustainability.

The clinic was offered one day a week for a six-month period from December 2022 to June 2023. During the clinic, an appropriately trained clinic nurse attended secure locations within the correctional centre that were near the incarcerated population’s housing areas. From these secure locations, the nurse offered one-on-one confidential STI testing to all incarcerated people living in the nearby area. Taking the clinic to the incarcerated people negated the need for movement within the centre and reduced additional pressure on an already overcrowded medical centre. The clinic nurses then attended the housing areas in person, announced that they were conducting STI testing and brought any volunteers back to the secure location.

Information about the satellite STI clinic was disseminated through presentations to potential participants who were then informed of the weekly and voluntary nature of the clinic. The clinics involved completing an adaption of O'Byrne et al’s (2016) self-directed sexual health history questionnaire, including the provision of sexual health education (World Health Organization, 2019), and tailored to the carceral populations’ individual needs. Testing aligned with the Australian STI management guidelines for people in custodial settings by testing for chlamydia, gonorrhoea, syphilis, HIV, HCV and HBV, however, based on an assessment of risk within the testing site and paucity of resources, testing for hepatitis A was not conducted (Australasian Society for HIV Viral Hepatitis and Sexual Health Medicine, 2021a). Subsequently, trichomoniasis was included in clinic testing due to demographic risk factors, including age and ethnicity (Australasian Society for HIV Viral Hepatitis and Sexual Health Medicine, 2024b). Blood and urine specimens were taken, and if clinically indicated, ulcer, penile, rectal and/or oral swabs were offered (Australasian Society for HIV Viral Hepatitis and Sexual Health Medicine, 2021a; Australasian Society for HIV Viral Hepatitis and Sexual Health Medicine, 2024a; Australasian Society for HIV Viral Hepatitis and Sexual Health Medicine, 2021b; Australasian Society for HIV Viral Hepatitis and Sexual Health Medicine, 2024b). When concluding each encounter, an open-ended patient satisfaction survey was provided verbally by a clinic nurse with responses documented in the patient’s medical chart *verbatim*. Any participants who tested positive to an STI/BBV were re-called to the onsite medical centre to be informed of their diagnosis, receive treatment, discuss contact tracing, receive further education and be informed of their next follow-up appointment.

## Methods

### Study design

A cohort study through a retrospective medical chart audit was conducted between October 2023 and March 2024.

### Setting

The setting was a maximum-security men’s correctional centre in Queensland, Australia with approximately 1,550 incarcerated adult men, including a small cohort of transgender women. The centre has two 24-hour medical centres, operated by a 42-person nursing team. The nursing team includes a full-time BBV nurse who is primarily focused on HCV testing, treatment and follow-up. Any positive HCV diagnosis found in the satellite STI testing clinic were referred to the BBV nurse for treatment and follow-up.

Prior to this satellite clinic, incarcerated people accessed sexual health services by submitting a health service request form, and unless urgent, were waitlisted for review before being assessed in the correctional facility’s medical centre, taking approximately three-months from request to assessment.

### Participants

All men, and transgender women, over the age of 18, residing in the correctional centre at the time of the study were eligible to participate. A sample size calculation was not conducted, rather a convenience sample was used. The estimated increases/decreases in percentages were estimated to be clinically significant.

### Data sources/measurement

Satellite STI testing clinic attendance rates were measured by the number of participants who attended the clinic, divided by the total number of eligible participants. Clinic attendance was due to the opportunistic offering of testing for both symptomatic and asymptomatic patients. Treatment rates were measured by the number of participants treated three-months prior to the intervention, divided by the total number of participants in the intervention. Time to treatment was measured by the time between testing and treatment three-months prior to the intervention, divided by the time between testing and treatment during the intervention. Reinfection rates were measured by the number of reinfections three-months prior to the intervention, divided by the number of reinfections during the intervention. Participants were considered to be reinfected if they were diagnosed with an STI/BBV after receiving effective treatment, or if diagnosed with an STI/BBV after testing negative to any other STI/BBVs.

Follow-up rates were measured by the number of follow-up tests three-months prior to the intervention, divided by the number of follow-up tests during the intervention. Follow-up testing was offered to participants after receiving treatment for an STI/BBV detected through the clinic. Satisfaction with the clinic service was measured by responses to a satisfaction survey using numeric responses to “On a scale of 0–10, where 10 means “Most Likely to Recommend” and 0 means “Not at all Likely to Recommend,” how likely would you be to recommend the clinic to other incarcerated people?”. Prior to the intervention, satisfaction surveys were not conducted as this is not a standard procedure for medical centre attendance. Sustainability was considered to be organisational and financial change to support the continuation of an intervention (Kilbourne *et al.*, 2007). In this intervention, the level of sustainability was measured by the continuation of the intervention post study funding.

### Bias

The retrospective medical chart audit of this intervention was conducted independently by two members of the research team (AB and ABr). To ensure inter-rater reliability, all medical charts for those that participated in the intervention were accessed independently by each research team-member using consistent and pre-agreed upon terminology, many of which were yes/no responses, and any disagreement in findings were discussed between the two independent researchers until a consensus was reached.

### Data collection

This study was approved by the Townsville Institute of Health Research & Innovation (TIHRI) HREC/2023/QTHS/97545, and the University of Southern Queensland Human Research Ethics Committee ETH2024-0346. Public Health Act (PHA) approval PHA97545 was also granted to access the retrospective medical chart audit data.

### Data analysis

Primary outcomes included increased attendance rates, increased STI/BBV treatment rates, reduced time to treatment, decreased reinfection rates, increased follow-up rates and high levels of client satisfaction achieved through a rating out of 10. In Table 1, variables were grouped into categories for clarity including demographic data, symptomology, pathology, previous diagnosis, reason for attending the satellite clinic, risk-behaviours, vaccination history and education provided in the clinic encounter. Table 2 presents outcome data variables from three-months prior to the program, during the satellite STI testing clinic program and from three months post the program.

**Table 1:** Demographic information, symptomology, testing methods used, previous diagnosis and reason for testing, risk behaviours, vaccination history and education provided for the study population, n = 242


		Mean	SD	Median	Range	N (%)
*Demographic data*						
Age		34.86	9.21	33.00	57	242 (100)
	*Total*					*242 (100)*
Ethnicity	Australian aboriginal					64 (26.4)
	Australian aboriginal and Torres strait islander					6 (2.5)
	Torres strait islander					3 (1.2)
	Australian					151 (62.4)
	Other countries of origin					18 (7.5)
	*Total*					*242 (100)*
Sex assigned at birth	Male					242 (100)
	Female					-
	*Total*					*242 (100)*
Symptomology						
Symptoms (At the time of clinic attendance)	No symptoms					228 (94.2)
	Discharge					-
	Lesions					5 (2.1)
	Penile pain					-
	Testicular pain					1 (0.4)
	Rectal pain					-
	Urinary pain or dysfunction					8 (3.3)
	Chancre					1 (0.4)
	Other symptoms (blood in semen)					1 (0.4)
	*Total*					*242 (100)*
Pathology						
	Blood					238 (98.3)
	Urine					233 (96.3)
	Penile swab					3 (1.2)
	-Declines					8 (3.3)
	Throat swab					2 (0.8)
	-Declined					4 (1.7)
	Rectal swab					3 (1.2)
	-Declined					4 (1.7)
	Ulcer swab					4 (1.7)
	-Declined					–
	*Total*					*242 (100)*
	*Missing system*c					-
Previous diagnosis						
Previous diagnosis (Patient history)	HCV					1 (0.4)
	Chlamydia					44 (18.2)
	Gonorrhoea					11 (4.5)
	Syphilis					4 (1.7)
	HSV					10 (4.1)
	Genital warts (HPV)					1 (0.4)
	More than one previous diagnosis					11 (4.5)
	YES					1 (0.4)
	*Total*					*83 (34.3)*
	*Missing system*					*159 (65.7)*
Timeframe of previous diagnosis in years	Years	6.94	6.38	4.24	33.08	82 (33.9)
	*Total*					*82 (33.9)*
	*Missing system*					*160 (66.1)*
Reason for attendingthe satellite clinic						
Reason for attending the satellite clinic	Symptomatic					
	Yes					15 (6.2)
	No					226 (93.4
					
	*Total*					*241 (99.6)*
	*Missing system*					*1 (0.4)*
	Convenience					
	Yes					225 (93.0)
	No					16 (6.6)
	*Total*					*241 (0.4)*
	*Missing system*					*1 (0.4)*
Risk behaviours						
Timeframe of last sexual active in years	Years	3.04	2.54	2.22	25.34	239 (98.8)
	*Total*					*239 (98.8)*
	*Missing system*					*3 (1.2)*
Type of sexual activity during last sexual encounter	Anal, vaginal and oral					58 (24)
	Vaginal					67 (27.7)
	Oral and vaginal					97 (40.1)
	Oral and MSM					1 (0.4)
	Oral					6 (2.5)
	MSM					1 (0.4)
	Oral, anal and MSM					3 (1.2)
	Oral and anal					2 (0.8)
	Anal					1 (0.4)
	Vaginal and anal					3 (1.2)
	*Total*					*239 (98.8)*
	*Missing system*					*3 (1.2)*
Protection used during last sexual encounter	Yes					18 (7.4)
	No					220 (90.9)
	*Total*					*238 (98.3)*
	*Missing system*					*4 (1.7)*
Type of protection used during last sexual encounter	Condom					3 (1.2)
	Bread bag					1 (0.4)
	Not stated					14 (5.8)
	*Total*					*18 (7.4)*
	*Missing system*					*224 (92.6)*
Ever participated in IVDU	Yes					155 (64)
	No					85 (35.1)
	*Total*					*240 (99.2)*
	*Missing system*					*2 (0.8)*
Received a prison tattoo and/or piercing	Yes					120 (49.6)
	No					120 (49.6
	*Total*					*240 (99.2)*
	*Missing system*					*2 (0.8)*
Other risk behaviour stated by participants	Kites					7 (2.9)
	Sex with other incarcerated people					3 (1.2)
	High level drug use					2 (0.8)
	*Total*					*12 (5)*
	*Missing system*					*230 (95)*
Vaccination history						
Hepatitis B vaccinated	Yes					138 (57)
	No					69 (28.5)
	Unsure					26 (10.7)
	Undergoing vaccination					1 (0.4)
	*Total*					*234 (96.7)*
	*Missing system*					*8 (3.3)*
Education provided						
Education provided during the satellite clinic encounter	STI risk activity education					
	YES					239(98.8)
	*Total*					*242(100)*
	*Missing system*					*3 (1.2)*
	STI prevention education					
	YES					221(91.3)
	NO					16 (6.6)
	*Total*					*237 (97.7)*
	*Missing system*					*5 (2.1)*
	Other education provided					
	Educational handouts					131(54.1)
	*Total*					*242 (100)*
	*Missing system*					*111 (45.9)*

**Source(s):** Table by authors

**Table 2:** Outcome data including attendance rates, treatment rates, time to treatment, follow-up rates, reinfection rates and client satisfaction, *n* = 242

		Three months prior to the program (Medical centre)				Satellite STI testing clinic program (Mobile clinic)				Absolute risk difference	Three months post the program(Medical centre)
Outcome data	Responses	*N* (%)				*N* (%)				Relative risk	*N* (%)
Attendance rate	Yes	32 (13.2)				242 (100)					35 (14.5)
	No	177 (73.1)				-					178 (73.6)
	Missing charts	33 (13.6)				-					29 (12)
	*Total:*	*242 (100)*				*242 (100)*				*6.531 (95%CI:4.748–8.984)*	*242 (100)*
	*STIs diagnosed on attendance:*										
	HCV	10 (4.1)				44 (18.2)					-
	Syphilis	1 (0.4)				-					-
	Chlamydia	-				1 (0.4)					-
	HSV 2	-				1 (0.4)					-
	Trichomoniasis	-				1 (0.4)					-
	HBV	-				1 (0.4)					-
	HCV and chlamydia	-				1 (0.4)					-
	HCV and HSV 2	-				2 (0.8)					-
	Chlamydia and trichomoniasis	-				1 (0.4)					-
	Missing charts	6 (2.5)				-					-
	*Total:*	*17 (7)*				*52 (21.5)*				*0.62 (95% CI:0.366–1.068)*	
	*Missing system:*	*225 (93)*				*190 (78.5)*					
*Treatment rate*	Yes	10 (4.1)				41 (16.9)					-
	No	14 (5.8)				189 (78.1)					-
	Discharged prior to treatment					4 (1.7)					-
	Declined treatment					2 (0.8)					-
	On treatment at the time of testing					3 (0.8)					-
	Cleared naturally					1 (0.4)					-
	Ineligible for treatment					1 (0.4)					-
				
	Missing charts	6 (2.5)									-
	Discharged prior to treatment	1 (0.4)									
				
	*Total:*	*31 (12.8)*				*241 (99.6)*				*0.867(95% CI:0.686–1.096)*	*-*
	*Missing system*	*211 (87.2)*				*1 (0.4)*					*-*
	Total number diagnosed that received treatment:										
	HCV	9 (3.7)				34 (14)					-
	Syphilis	1 (0.4)				-					-
	Chlamydia	-				1 (0.4)					-
	HSV 2	-				1 (0.4)					-
	Trichomoniasis	-				1 (0.4)					-
	HBV	-				-					-
	HCV and chlamydia	-				1 (0.4)					-
	HCV and HSV 2	-				-					-
	Chlamydia and trichomoniasis	-				1 (0.4)					-
	Declined treatment for HCV, but accepted for HSV2	-				1 (0.4)					-
	HCV treated. No immediate treatment required for HSV2	-				1 (0.4)					-
	Missing charts	6 (2.5)				-					-
	*Total:*	*16 (6.6)*				*41 (16.9)*					-
	*Missing system:*	*226 (93.4)*				*201 (83.1)*					-
*Time to treatment*		Median	Range	Mean	Std. Dev	Median	Range	Mean	Std. Dev		
		35	132	43.11	36.77	49	167	54.62	42.06		-
*Follow-up rate*	Follow-up testing accepted:										
	Yes:	5 (2.1)				24 (9.9)					-
	No	17 (7.0)				1 (0.4)					-
	N/a Discharged	-				13 (5.4)					-
	N/a Transferred	-				4 (1.7)					-
	Missing charts	6 (2.5)				2 (0.8)					-
	*Total:*	*28 (11.6)*				*44 (18.2)*				*3.000 (95% CI:1.196–7.526)*	-
	*Missing system:*	*214 (88.4)*				*198 (81.8)*					-
	Follow-up treatment complete										
	HCV	4 (1.7)				22 (9.1)					-
	Trichomoniasis	-				1 (0.4)					-
	Chlamydia	-				1 (0.4)					-
	Missing charts	6 (2.5)				-					-
	*Total:*	*10 (4.1)*				*24 (9.9)*					-
	*Missing system*	*232 (95.9)*				*218 (90.1)*					-
*Reinfection rate*	Was the patient reinfected?										
	Yes	2 (0.8)				22 (9.1)					-
	No	18 (7.4)				194 (80.2)					-
	Missing charts	6 (2.5)				26 (10.7)					-
	*Total:*	*26 (10.7)*				*242 (100)*				*0.935 (95 CI:0.235–3.713)*	*-*
	*Missing system:*	*216 (89.3)*				*-*					*-*
	Reinfection post treatment										
	HCV	2 (0.8)				15 (6.2)					-
	HSV1	-				1 (0.4)					-
	HSV2	-				3 (1.2)					-
	Gonorrhoea	-				1 (0.4)					-
	Missing charts	6 (2.5)				1 (0.4)					-
	*Total*	*8 (3.3)*				*21 (8.7)*					-
	*Missing system*	*234 (96.7)*				*221 (91.3)*					-
*Satisfactionrating out of 10*	*Mean score out of 10*	*-*				*9.7(97)*					–

**Source(s):** Table by authors

Quantitative data were analysed using SPSS 24. Categorical data are presented as counts and percentages. For continuous data, means and standard deviations (Std. Dev) were used to summarise normally distributed data; medians and ranges summarised non-normally distributed data. Categorical data were analysed using Pearson’s Chi-square and Fisher’s exact test to consider associations between demographic characteristics such as IVDU and outcome variables such as reinfection rates. Relative Risk (henceforth RR) was also included with a value of >1 used to indicate an increase in likelihood of each outcome pre-intervention to during the intervention. All missing systems, and missing chart information were provided for transparency including loss to follow-up due to transfers to other facilities, and on discharge/parole. To create clarity around all missing data, “missing charts” and ‘missing systems’ were both recorded separately. *Missing systems* referred to data that were missing because it was either not recorded in the medical chart, or because a patient refused/preferred not to provide information verbally. *Missing charts* referred to participant medical charts for which the research team was unable to access, such as charts from participants who had transferred to other facilities, where no ethical approval was granted. This lack of access to medical charts only affected the data collected three-months prior to the intervention, reinfection rates during the intervention and data collected three-months after the intervention.

## Result

A total of 242 participants, all assigned male at birth, attended 253 clinic visits, while 11 persons attended the clinic twice during the six-month period. Positive STI diagnosis were found in 52 participants. Notably, 44 of the 52 diagnoses were HCV, with 8 diagnoses being either Chlamydia, Trichomoniasis, HCV, HBV, HSV2 or a combination of these. No participants were diagnosed with gonorrhoea. Of the 52 participants who received a positive STI diagnosis, 41 received treatment and 11 participants did not. Time to treatment was assessed for 42 participants, where 24 received follow-up testing, and 20 participants did not. Twenty participants were reinfected with an STI post-treatment or after testing negative to an STI in the clinic (see Figure 1).

**Figure 1:**
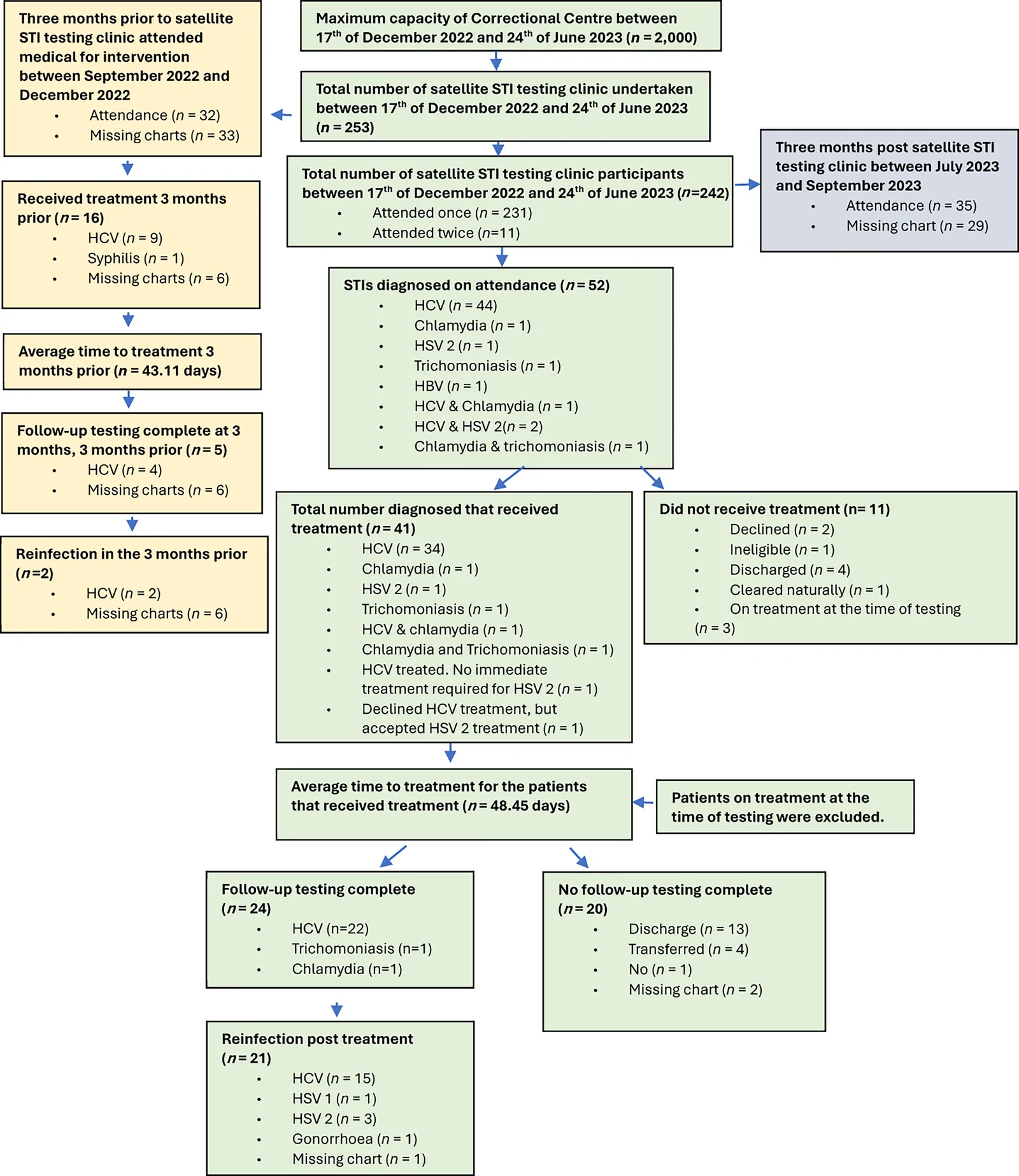
Flow chart demonstrating the number of participants at each stage of the study **Source:** Figure by authors

The mean age was 34 years (Std. Dev 9.21), and 21 ethnicities were identified with 62.4% self-identifying as Australian, 26.4% as Aboriginal, 1.2% as Torres Strait Islander and 2.5% as both Australian Aboriginal and Torres Strait Islander with the remaining participants from African, Asian and European nations (see Table 1). Of the total number of clinic attendees, 94.2% stated they were asymptomatic at the time of testing.

Pathological testing was conducted aligning with national guidelines (Australasian Society for HIV Viral Hepatitis and Sexual Health Medicine, 2021a; Australasian Society for HIV Viral Hepatitis and Sexual Health Medicine, 2021b; Australasian Society for HIV Viral Hepatitis and Sexual Health Medicine, 2024a; Australasian Society for HIV Viral Hepatitis and Sexual Health Medicine, 2024b). Blood samples were provided by 98.3% of participants, and 96.3% provided urine specimens. Swabs were offered when clinically indicated [e.g. men who have sex with men (MSM) patients], however, 8 of 11 participants offered penile swabs declined. One or more previous STIs diagnoses were reportedly diagnosed in the last 6.94 years and were found in 33.9% of participants. More than half reported a previous diagnosis of chlamydia. Reasons for testing included being symptomatic (6.2%) and convenience (93%).

More than 90% of participants reported to not using protection in their last sexual encounter, where multiple participants disclosed partaking in anal sex and/MSM 28.4%, further 64% disclosed that they had ever participated in IVDU and 49.6% had received a prison tattoo/piercing. Additional risk behaviours were captured in the “other” section, where three reported having sex with other incarcerated people, two reported high-level drug use (relating to self-use) and seven disclosed placing small handwritten notes “Kites” underneath the foreskin for movement around the centre which were passed from one person’s foreskin to another’s. Of the participants, 57% were HBV vaccinated; all unvaccinated patients were offered vaccination referral. Education provided during the satellite clinic encounter included STI risk education, which was provided to 98.8% of the participants, where 91.3% received STI prevention education, specifically, and 54.1% were given educational handouts. All outcome findings are represented in Table 2, including any missing charts, and missing systems for transparency.

### Outcome data

#### Attendance rates.

Attendance rates increased from 32/242 (13.2%) to 242/242 (100%), pre-intervention to intervention, respectively. Comparing attendance rates pre-intervention to intervention, the RR of 6.53 was 60.8% (95% CI:4.74–8.98). Demonstrating participants were 6.5 times more likely to attend due to the satellite STI testing clinic.

#### STI diagnosis on attendance.

Diagnosis of STIs/BBVs increased from 10/242 (4.13%) to 52/242 (21.5%) pre-intervention to during the intervention, respectively. When comparing STI diagnosis rates pre-intervention to intervention, the RR of 0.62 was 23% (95% CI: 0.366–1.068), showing 38% less people were diagnosed with an STI prior to the clinic intervention. A statistically significant association between STI diagnosis and IVDU (19.431, *p* = <0.034), symptomology (33.250, *p* = <0.001) and convenience (30.141, *p* = <0.001) was found.

### Treatment rates

Treatment rates increased from 10/242 (4.1%) to 41/242 (16.9%) pre-intervention to intervention respectively. Treatment was not provided to 11/242 (4.54%) due to two participants declining treatment, one being ineligible for treatment, four participants being discharged from the centre one clearing the STI naturally and three were on treatment at the time of testing. Comparing treatment rates pre-intervention to intervention, the RR of 0.86 was 81% (95% CI: 0.68–1.09), demonstrating that participants in the intervention were 14% less likely to be treated. A statistically significant association between treatment rates and IVDU (27.161, *p* = <0.001), and prison tattoos/piercing (11.868, *p* = 0.014) was evident.

### Time to treatment

Time to treatment increased from 43.11 (Std. Dev 36.77) mean days pre-intervention to 54.62 (Std. Dev 46.64) mean days during the intervention (excluding three people already on treatment at the time of participation).

### Follow-up rates

Follow-up rates increased from 5/242 (2.1%) pre-intervention to 24/242 (9.9%) during the intervention, due to participants being discharged 13/242, (5.4%), transferring to different centres 4/242 (1.7%), missing charts 1/242(0.4%) and declining the follow-up testing 1/242 (0.4%). Comparing follow-up rates pre-intervention to intervention, the RR of 3.00 was 44.4% (95% CI: 1.19–7.52), highlighting participants were three times more likely to receive follow-up testing due to the intervention.

### Reinfection rates

Reinfection rates increased from 2/242 (0.8%) pre-intervention to 21/242 (8.7%) during the intervention. Comparing reinfection rates pre-intervention to intervention, RR of 0.93 was 9.4% (95% CI: 0.23–3.71) which indicated the risk of reinfection was reduced by 7% due to the intervention. A significant statistical association was found between reinfection rates and IVDU (16.579, df^2^, *p* = <0.001) and prison tattooing/piercing (12.377, df^2^, *p* = <0.002).

### Satisfaction rating out of 10

During the intervention a mean score of 9.7/10 was achieved for the client satisfaction survey highlighting that participants were likely to recommend the clinic to other incarcerated people. Satisfaction scores were introduced in week five of the intervention, therefore all missing data (*n* = 64) were not included when calculating this average. Attendance rates showed no significant difference three-months pre- and post-intervention when no structured intervention was being implemented, suggesting that without the satellite clinic, STI/BBV testing was less opportunistic and less likely to occur. Further financial support was provided by a new funding body “Communicable Diseases Branch, Queensland Public Health and Scientific Services Division - Queensland Health” to continue the satellite STI testing clinics for an additional 12 months. In addition, the intervention was included as a component of the Queensland Syphilis Action Plan for 2023–2028 Section 2.4, Item 7 (Queensland Health, 2023) showing great promise for sustainability, and future upscale of this intervention across all correctional centres in Queensland.

## Discussion

The aim of this study was to determine if a nurse-led satellite STI clinic increased STI/BBV testing and detection in a male carceral setting. Findings from this study indicate that the satellite STI clinic was successful through an increase in STI/BBV attendance/testing, treatment and follow-up rates. Furthermore, attendance rates were strongly associated with convenience which aligns with existing literature asserting the effectiveness of mobile healthcare. For example, Hesse *et al* (2015) conducted STI testing using a mobile van to deliver testing to people at high-risk of STI/s, and found the method both feasible and acceptable. This study also found attendance rates improved because of convenience, concluding that to improve accessibility, future practice could benefit from including STI/BBV testing through mobile settings for high-risk populations, such as incarcerated populations.

Whilst the aim of the study was to examine STI/BBV testing and detection, among the participants who tested positive for an STI/BBV, the majority tested positive for HCV. This is unsurprising due to HCV being prevalent in Australian incarcerated settings (Bah *et al.*, 2024). However, it does indicate that greater efforts in screening and treatment for HCV within prison settings are required to prevent transmission and illness trajectory. Considering the HCV diagnosis rate, it is also unsurprising that IVDU and STI/BBV diagnoses were found to be statistically significant. Furthermore, a notable increase in reinfection rates were found from pre-intervention to during the intervention, where both the upscale in testing, and the statistical significance with IVDU and prison tattoo informed this increase, also found in other research (Hajarizadeh *et al.*, 2021). A significant increase in STIs/BBV was also found with the implementation of the clinic, however, RR showed participants were less likely to be diagnosed with an STI/BBV as a result of attending the satellite STI testing clinic. A potential reason for this higher level of STI/BBV diagnosis prior to the intervention was due to participants presenting because they were symptomatic.

Eleven of the 32 participants who presented prior to the intervention had a positive STI/BBV diagnosis. These findings demonstrate the difference between the previous process for sexual health testing (by submitting a health service request form, waitlisted for review unless urgent, before being assessed in the medical centre), and opportunistic testing. Pimenta *et al.* (2003) had similar findings when they conducted opportunistic testing for chlamydia through urine testing in both primary and secondary healthcare environments. Their study found that opportunistic testing succeeded in obtaining a sizable population coverage particularly in asymptomatic patients and was considered acceptable and practical (Pimenta *et al.*, 2003). Regrettably, in the current study data on patient symptoms as a component of the pre-intervention/baseline data were not collected. Future research would benefit from the inclusion of symptomology in the pre-intervention/baseline data in carceral settings.

Treatment was provided to 79% of those with positive STIs/BBV diagnoses. Similar findings have been reported from a universal HCV testing program in combination with telemedicine across prisons in England, with 71% commencing HCV treatment whilst incarcerated, and of this number only 73% completed treatment whilst incarcerated due to being discharged or transferred to alternative facilities (Morey *et al.*, 2019). Whilst patients were referred for treatment and/or follow-up in the community, the research team was unable to access any further medical information on these patients once they had been discharged/paroled. This suggests that the barriers to treatment within this study are experienced in other correctional centres too. However, considering the increase in treatment rates, which may not have occurred without the clinic, this was deemed a positive outcome. Similarly, it was found that follow-up rates were negatively impacted by participants being transferred or discharged prior to follow-up, yet the increase in follow-up rates as a beneficial outcome of the clinic was also noted. Other research has found almost 50% of their participants did not receive follow-up for HCV in Australian prisons due to the transient nature of the testing population with discharge and transfers being a regular occurrence (Hajarizadeh *et al.*, 2021). These results highlight a barrier affecting the incarcerated population’s ability to receive sexual health care that promotes positive wellbeing.

This study also found a delay in time to treatment (median 49-days), similar to previous research by Mohamed et al (2020) where it took a median of 36 days using conventional screening from diagnosis to treatment for HCV among incarcerated people. Whilst our findings differ from those which are found in other research, where time to treatment for HCV was as short as 6 days from diagnosis to commencement of antiviral therapy (Sheehan *et al.*, 2023) anecdotal limitations, such as HCV participants from the present study, choosing to accept treatment when they were ready severely impacted time to treatment findings in this study. Future research would benefit from the inclusion of these factors when calculating the time to treatment.

The level of client satisfaction was high with most participants likely to recommend the clinic to other incarcerated people. Measuring participant satisfaction aligns with national prison health initiatives seeking to improve processes to (1) access and request health services; (2) timely response times; and (3) communication to improve client satisfaction (Queensland Government, 2020). Due to the absence of client satisfaction data prior to the present intervention, resulted in the inability to make comparisons, however, a 97% client satisfaction rate in favour of the clinic’s presence and service, was a significant outcome.

### Limitations

There were multiple limitations to this study including the inability to follow-up on 33-participants as they had transferred to another carceral centre. Whilst PHA approval was granted to access the participant medical charts, site specific approval only applied to the one specific research site, so when people were transferred, the research team was unable to access data held at other correctional centres. Future research may consider the appropriate avenues to continue follow-up across centres. In addition, although patients were able to be tested in the satellite STI testing clinic, they were unable to receive treatment via this method. More specifically, potential treatments such as intramuscular injections were required to be given within the onsite medical centre due to enhanced privacy. Finally, due to the small sample, these study findings are not generalisable, however, considering the success in achieving increased STI testing attendance rates, treatment rates, follow-up rates and high satisfaction rates with the satellite STI testing clinic, replication of the nurse-led model is highly recommended.

## Conclusion

This study brings to light the importance of regular STI/BBV testing clinics among incarcerated adults to improve attendance rates, increase treatment rates, improve follow-up rates and to support high levels of client satisfaction. Future practice should incorporate satellite STI/BBV testing clinics to deliver regular STI/BBV testing as routine practice. Replication of the clinic could include further evaluation of the planning process to ensure that opportunities to collect data are not missed, especially when incarcerated people are transferred to other carceral centres. In addition, future research could focus on enhance treatment times to further improve the sexual and overall health outcomes among incarcerated adult populations.
